# Supplementary material for: Revealing cancer driver genes through integrative transcriptomic and epigenomic analyses with Moonlight
Source: PLoS Comput Biol. 2025 Apr 21;21(4):e1012999. doi: 10.1371/journal.pcbi.1012999 (PMC12058160; doi:10.1371/journal.pcbi.1012999)
Supplement: S2 Fig — Number of driver gene-drug interactions in (A) basal-like breast cancer, (B) lung adenocarcinoma, and (C) thyroid carcinoma found by querying DGIdb. The driver genes are stratified into OCGs and TSGs. The number of drug interactions is shown on the x axis and the driver genes are shown on the y axis. (PDF) [file pcbi.1012999.s002.pdf]

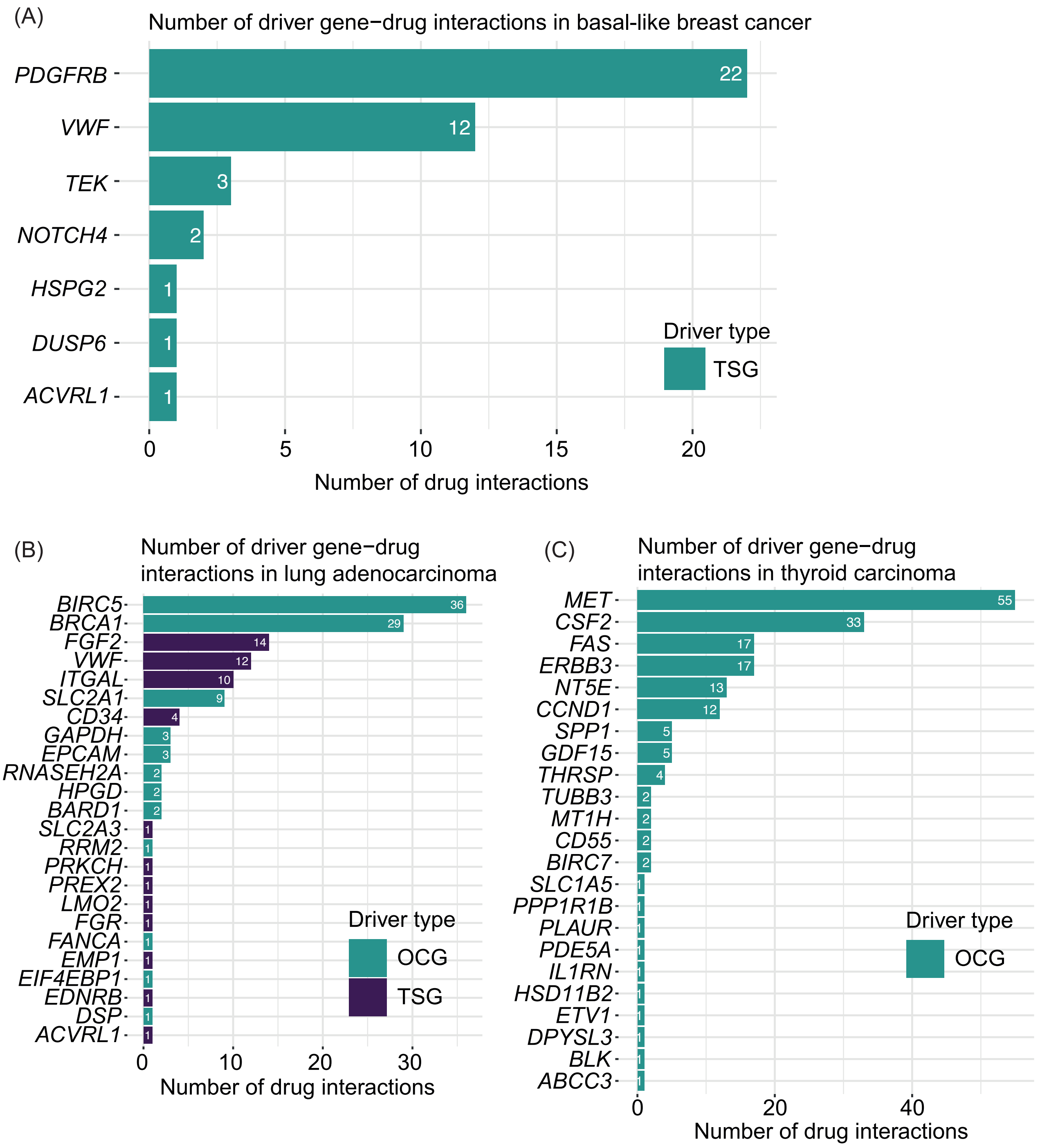

S2 Fig. Number of driver gene-drug interactions. Number of driver gene-drug interactions in **(A)** basal-like breast cancer, **(B)** lung adenocarcinoma, and **(C)** thyroid carcinoma found by querying DGIdb. The driver genes are stratified into OCGs and TSGs. The number of drug interactions is shown on the *x* axis and the driver genes are shown on the *y* axis.
