# Supplementary material for: Revealing cancer driver genes through integrative transcriptomic and epigenomic analyses with Moonlight
Source: PLoS Comput Biol. 2025 Apr 21;21(4):e1012999. doi: 10.1371/journal.pcbi.1012999 (PMC12058160; doi:10.1371/journal.pcbi.1012999)
Supplement: S2 Text — (PDF) [file pcbi.1012999.s004.pdf]

S2 Text

Results from driver-gene drug target analysis using a newer version of the DGIdb web API

Methods

In the manuscript, we presented results of the driver gene-drug target analysis that take advantage of the Drug-Gene Interaction Database (DGIdb) [1]. Originally, we had used an R package (rDGIdb) to perform our queries to DGIdb [2]. However, while testing our case studies for reproducibility at a later date, we found out that rDGIdb uses an old web API endpoint from DGIdb which appears to have been discontinued, and the package itself is currently scheduled for deprecation for the next release of BioConductor. Therefore, we have designed and used custom code to query DGIdb using a new and different API endpoint, using a query that would be as similar as possible to the one performed to the old web API.

In this document, we showcase the results we obtained using this newer web API version, to examine the impact of this change on our results.

Results

Number of driver gene-drug interactions using the updated version of DGIdb

In basal-like breast cancer, we originally identified seven TSGs documented to interact with drugs in DGIdb (S2A Fig). Using the updated database, we also find that these same seven TSGs interact with drugs listed in DGIdb (S3 Fig). We observe small differences in the number of drugs found to interact with these TSGs. The TSG *PDGFRB* is still the driver gene with the most drug interactions, which we originally found to be 22, which is now 23. Similar differences are found for the remaining six TSGs (S2 Fig A, S3 Fig).

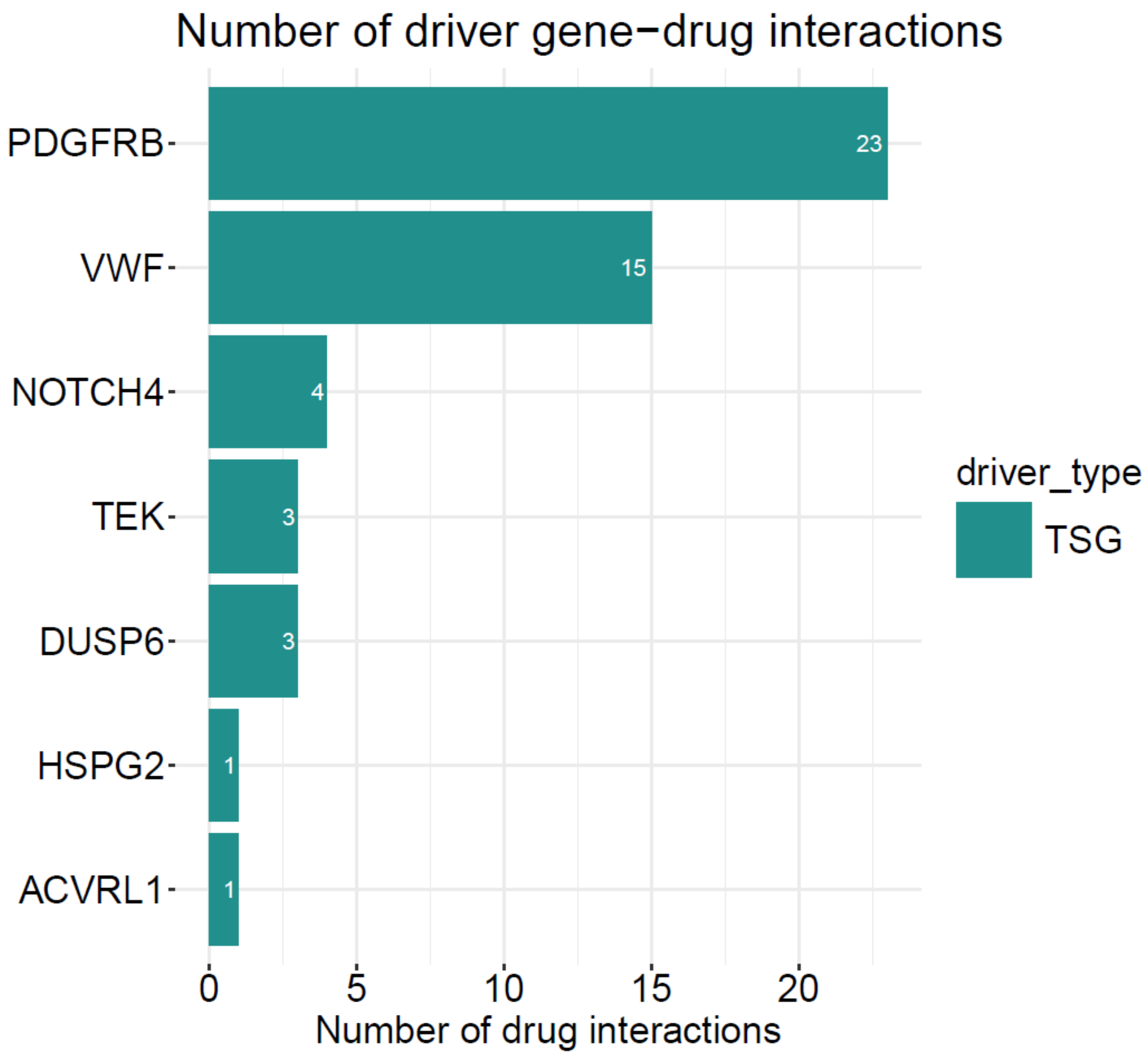

S3 Fig. Number of driver gene-drug interactions in basal-like breast cancer using the updated version of DGIdb.

In lung adenocarcinoma, we originally found 24 driver genes (12 OCGs and 12 TSGs) reported as drug targets (S2B Fig). In the updated results (S4 Fig), we found a slightly higher number of driver genes interacting with drugs, namely 32 driver genes (16 OCGs and 16 TSGs). Thus, 4 more OCGs (*CYP4B1*, *RUNX2*, *CXCR5*, *ABCC2*) and 4 more TSGs (*NCF2*, *FOXF1*, *FLI1*, *ALPL*) have been found to interact with drugs in the newer results. In the newer results, *BIRC5* is retained as the driver genes with the most drug interactions (57 interactions in the updated results compared to 36 interactions in our original results) (S2 Fig B, S4 Fig). All driver genes annotated with drug interactions in our original results (S2 Fig B) were retained in the updated results (S4 Fig). Overall, most of the driver genes had more drug interactions in the updated results compared to the original results, suggesting an expansion of gene-drug interaction data in DGIdb.

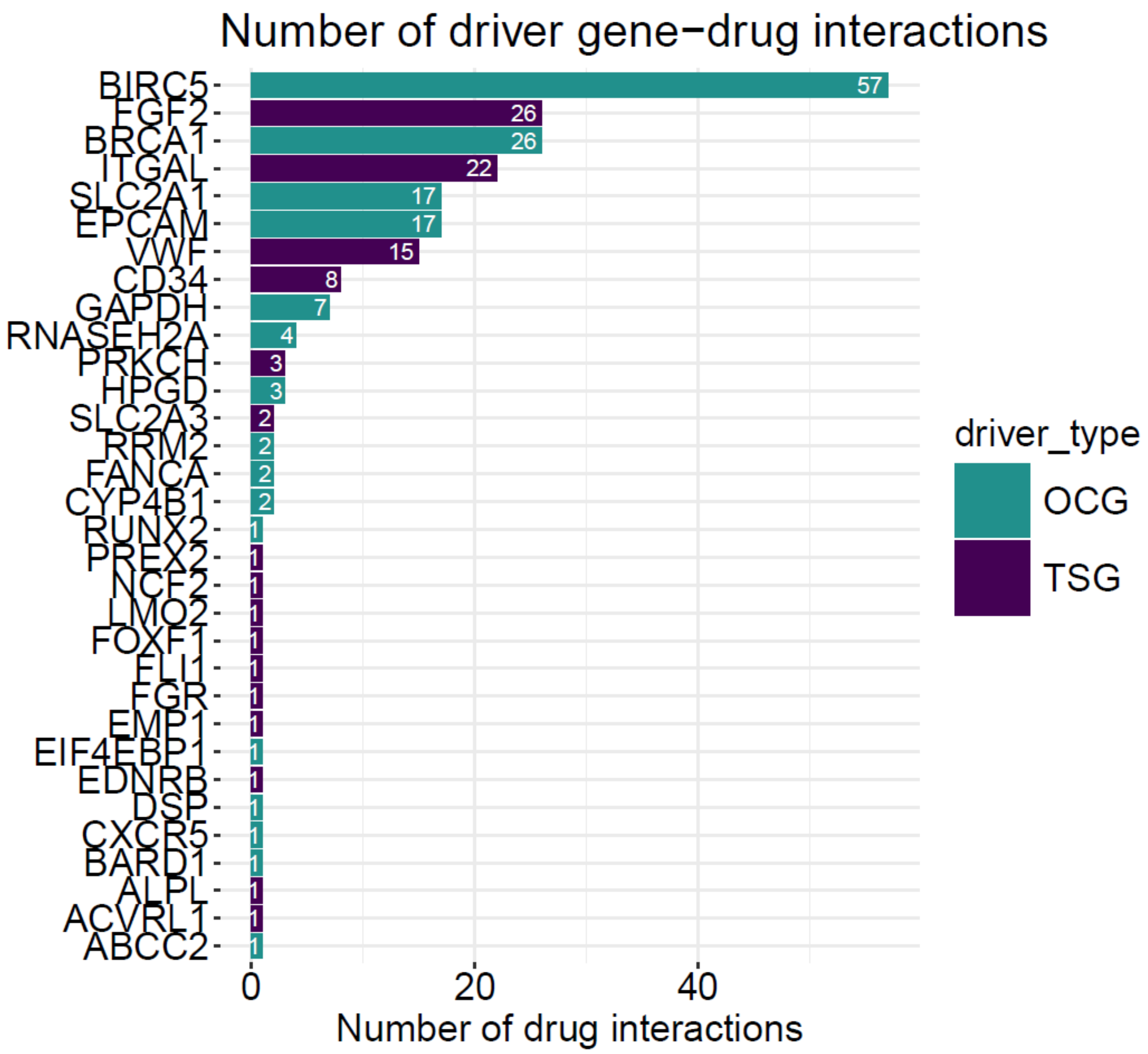

S4 Fig. Number of driver gene-drug interactions in lung adenocarcinoma using the updated version of DGIdb.

In thyroid carcinoma, we originally found 23 OCGs reported as interacting with drugs (S2 Fig C). In the updated results (S5 Fig), we found 33 OCGs interacting with drugs. All OCGs reported as interacting with drugs in the original results were retained in the updated results. Thus, we find an addition of 10 OCGs in the updated results which are *MUC1*, *IVL*, *NRIP1*, *HAVCR2*, *BHLHE40*, *PTGDS*, *MT1G*, *IL7R*, *IGFBP6* and *ALOX5AP*. In general, most of the driver genes had either more or the same number of drug interactions when comparing the original and updated results. The biggest differences between the updated and new results are the two OCGs with the largest number of interactions, *MET* and *CSF2*. These two OCGs are retained as the two OCGs with the largest number of interactions in the updated results, however, we found twice as many interactions of *CSF2* in the new results (60 interactions) compared to 33 interactions in the original results. *MET* has 35 interactions in the new results compared to 55 interactions in the original results (S2 Fig C, S5 Fig).

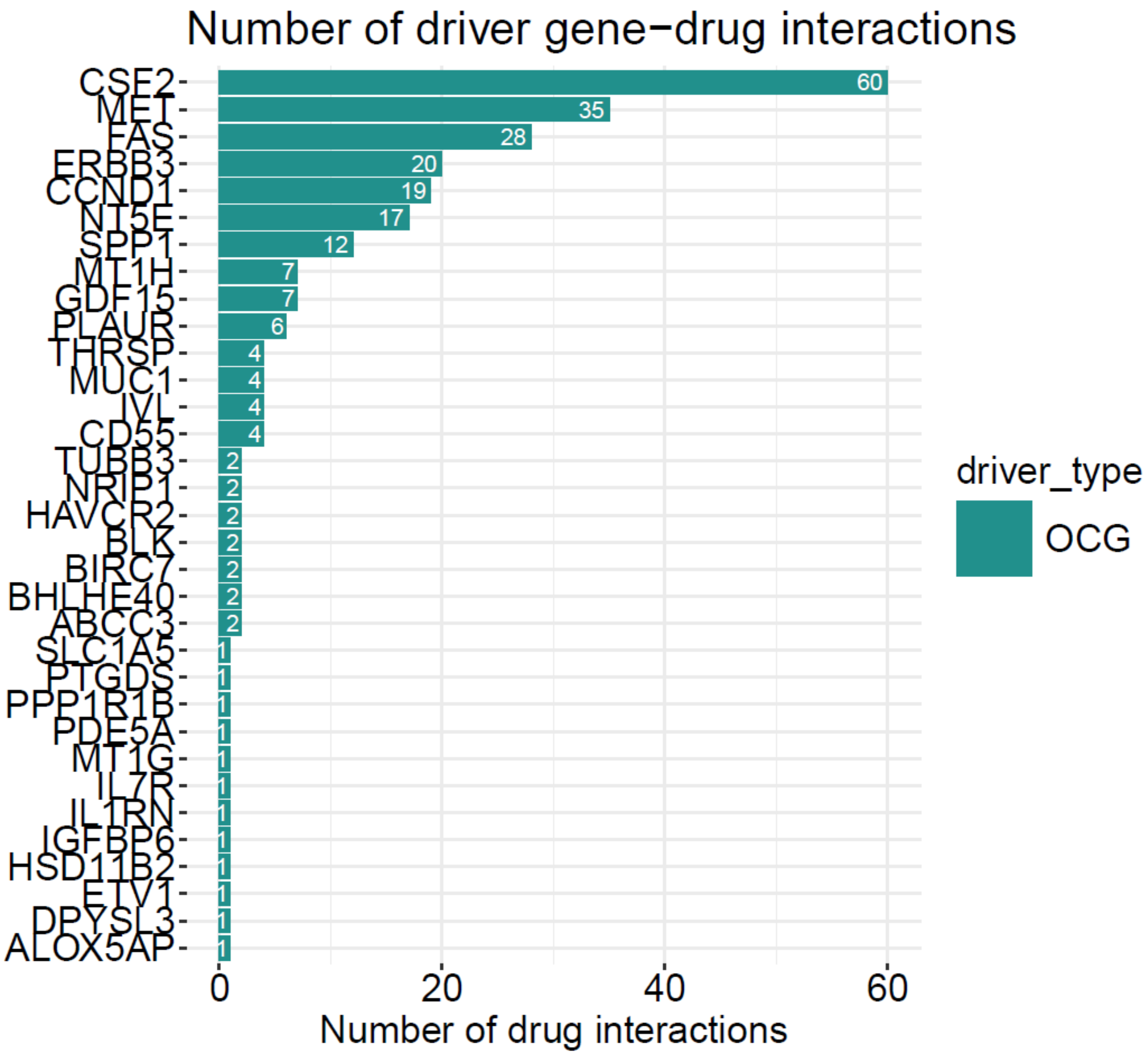

S5 Fig. Number of driver gene-drug interactions in thyroid carcinoma using the updated version of DGIdb.

Driver gene-drug interactions with known interaction type

In basal-like breast cancer, we originally found three TSGs (*TEK*, *PDGFRB*, and *NOTCH4*) for which the interaction type was known (Fig 5C). Using the updated version of DGIdb, these three TSGs were retained as driver gene-drug interactions where the interaction type was known with the addition of a fourth TSG, namely *ACVRL1* (S6 Fig). Comparing the results between the old and new version of DGIdb, we observe small differences in the results. E.g. the driver gene *TEK* was annotated as having two inhibitory interactions with REGORAFENIB and GLESATINIB in the original results (Fig 5C), and in the new results (S6 Fig), we find that *TEK* is annotated as having three inhibitory interactions with REGORAFENIB, GLESATINIB,

and CABOZANTINIB S−MALATE. Similarly, we also see small differences in the annotations when comparing the other TSGs (Fig 5C, S6 Fig).

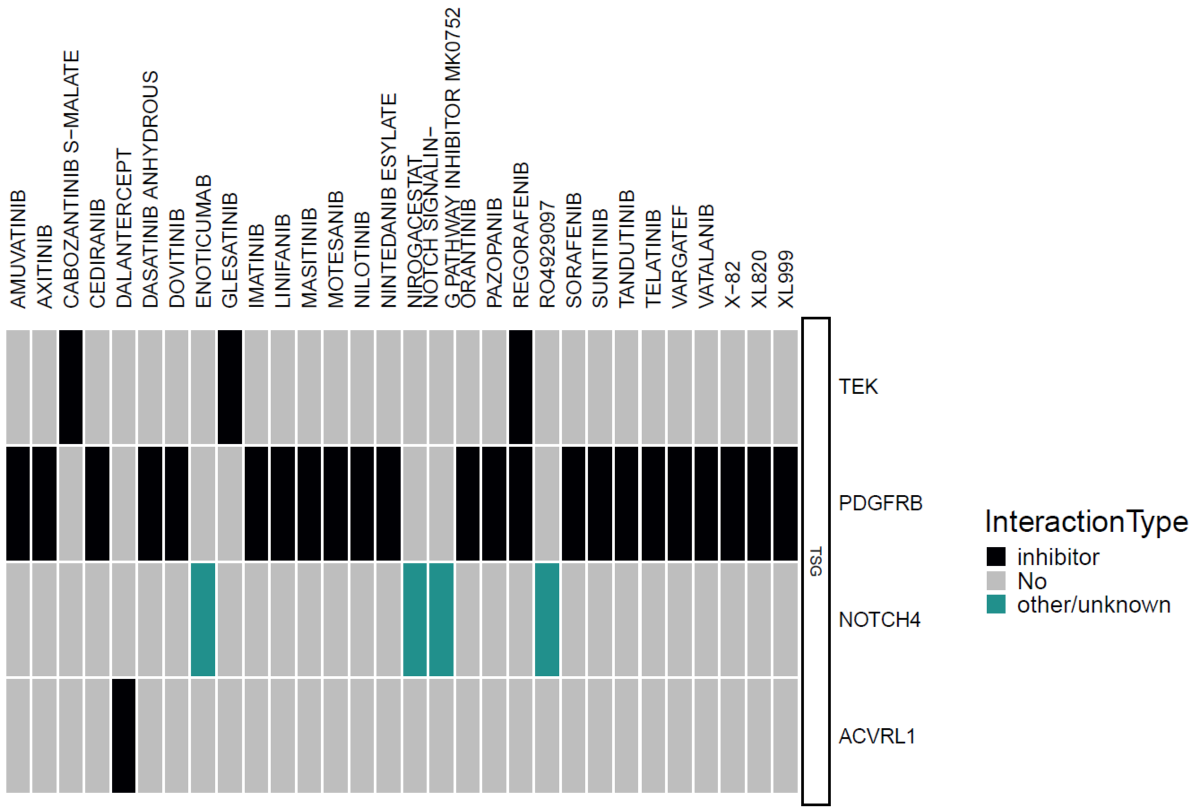

S6 Fig. Heatmap visualizing driver gene-drug interactions in basal-like breast cancer. Only those driver gene-drug interactions where the interaction type was known are included in the heatmap. The type of interaction is shown in different colors.

In lung adenocarcinoma, we originally found six driver genes (three OCGs and three TSGs), where the interaction type between gene and drug was known (Fig 5B). In the updated results (S7 Fig), we found that all three OCGs and one of the TSGs were retained in the updated results, namely OCGs *RRM2*, *EPCAM* and *BIRC5* and TSG *PRKCH*. Thus, the two TSGs found in the original results, *FGR* and *FGF2*, were not included in the updated results. However, another TSG was included in the updated results, namely *ACVRL1*. *FGR* and *FGF2* were however still found to interact with drugs as seen in S4 Fig, but their exclusion in S7 Fig might be attributed to a lack of annotation of the interaction type in the updated results from DGIdb. Comparing the original and new results, we observe that the OCG *RRM2* was found to interact with the inhibitor GEMCITABINE in the original results (Fig 5B), and in the updated results, this interaction is retained, and moreover, *RRM2* is found to interact with another inhibitor TRIAPINE in the updated results (S7 Fig). The interactions between *EPCAM* and SOLITOMAB and CATUMAXOMAB are retained in the

updated results. We found two new interactions of *BIRC5* in the updated results, and the inhibitory interaction between *PRKCH* and QUERCETIN is retained in the updated results.

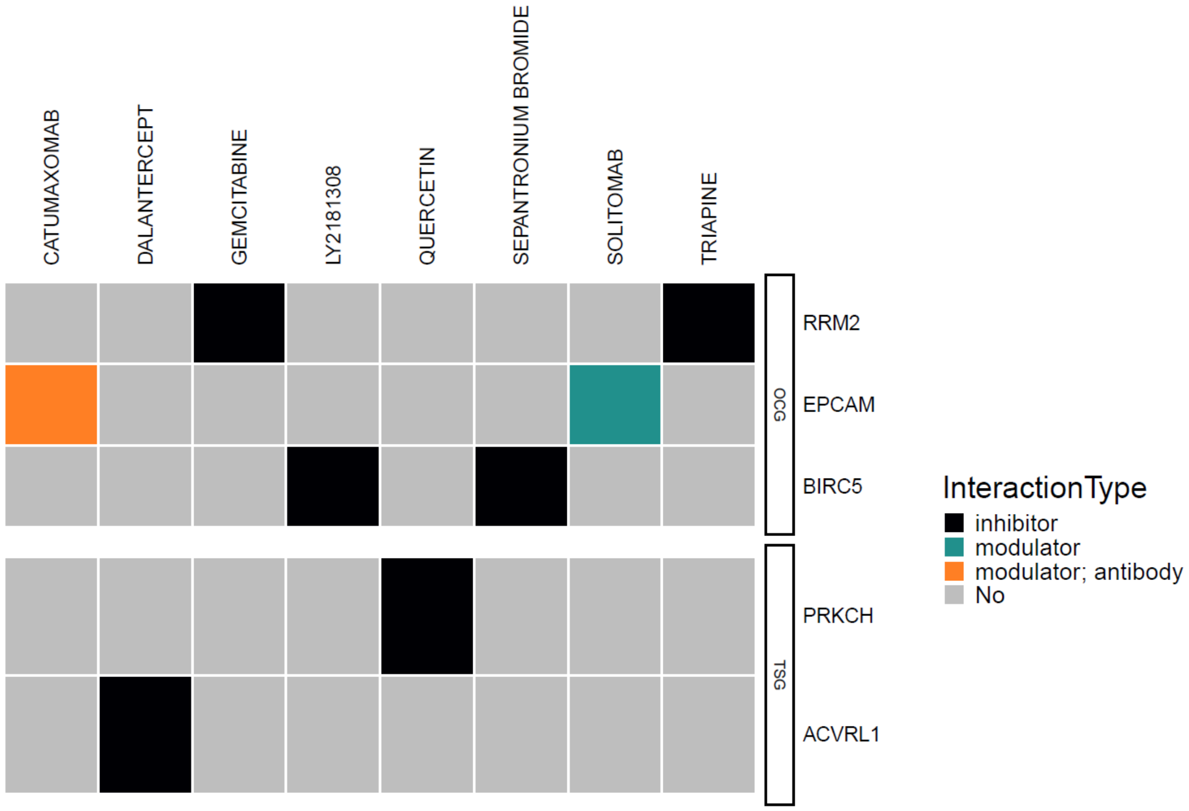

S7 Fig. Heatmap visualizing driver gene-drug interactions in lung adenocarcinoma. Only those driver gene-drug interactions where the interaction type was known are included in the heatmap. The type of interaction is shown in different colors.

In thyroid carcinoma, we originally found five OCGs where the interaction type between gene and drug was known (Fig 5D). In the updated results (S8 Fig), we found six OCGs where the interaction type between gene and drug was known. Four of the five OCGs in the original results were retained in the updated results, namely the OCGs *TUBB3*, *MET*, *ERBB3*, and *BIRC7*. Thus, the single interaction between the OCG *BLK* and NINTEDANIB in the original results was not retained in the updated results for drug-gene interactions with a known interaction type. However, *BLK* was still found to interact with two drugs in the updated results (S5 Fig), and thus, the lack of inclusion of *BLK* in S8 Fig might be attributed to a lack of annotation of the interaction type in the updated results. Additionally, two other OCGs were included in the updated results of drug-gene interactions with a known interaction type (S8 Fig) compared to the original results, namely *MUC1* and *CCND1*. Comparing the original and updated results, we found overall similar results. For

example, the inhibitory interaction between *TUBB3* and PACLITAXEL was retained in the updated results. Similarly, many of the interactions between drugs and *MET* and *ERBB3* were retained in the updated results.

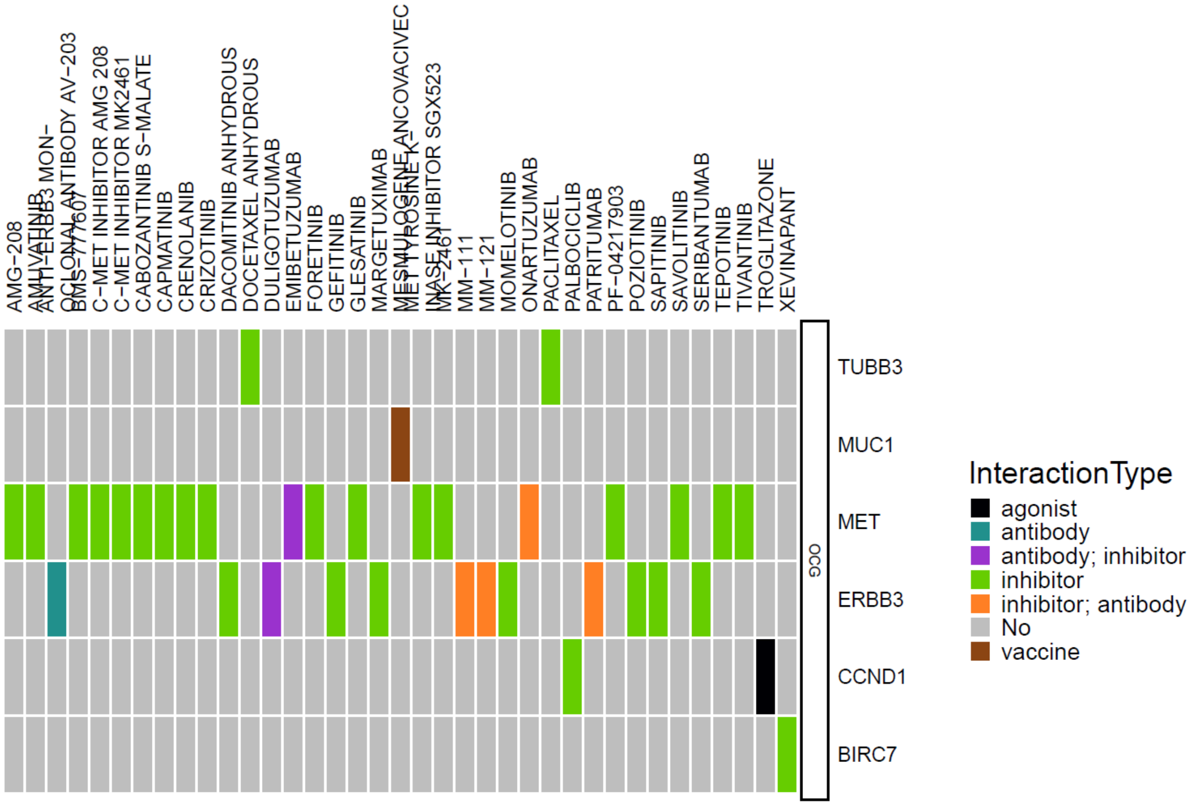

S8 Fig. Heatmap visualizing driver gene-drug interactions in thyroid carcinoma. Only those driver gene-drug interactions where the interaction type was known are included in the heatmap. The type of interaction is shown in different colors.

References

[1] DGIdb 5.0: rebuilding the drug-gene interaction database for precision medicine and drug discovery platforms. Cannon M, Stevenson J, Stahl K, Basu R, Coffman A, Kiwala S, McMichael JF, Kuzma K, Morrisey D, Cotto KC, Mardis ER, Griffith OL, Griffith M, Wagner AH. Nucleic Acids Research. 2024 Jan 5; doi: 10.1093/nar/gkad1040

[2] Genomic variant annotation workflow for clinical applications. Thurnherr T, Singer F, Stekhoven DJ, Beerenwinkel N. F1000Research. 2016; doi: 10.12688/f1000research.9357.2
