# Supplementary figures and images for: Revealing cancer driver genes through integrative transcriptomic and epigenomic analyses with Moonlight
Source: PLoS Comput Biol. 2025 Apr 21;21(4):e1012999. doi: 10.1371/journal.pcbi.1012999 (PMC12058160; doi:10.1371/journal.pcbi.1012999)

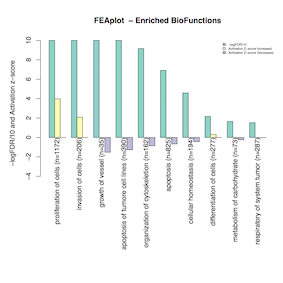

Supplement: S1 Data — (ZIP) [file pcbi.1012999.s005.zip › Moonlight2R-devel/vignettes/FEAplot.gif]

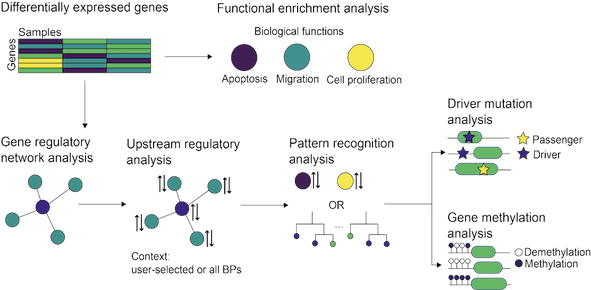

Supplement: S1 Data — (ZIP) [file pcbi.1012999.s005.zip › Moonlight2R-devel/vignettes/Moonlight2_pipeline_upd.png]

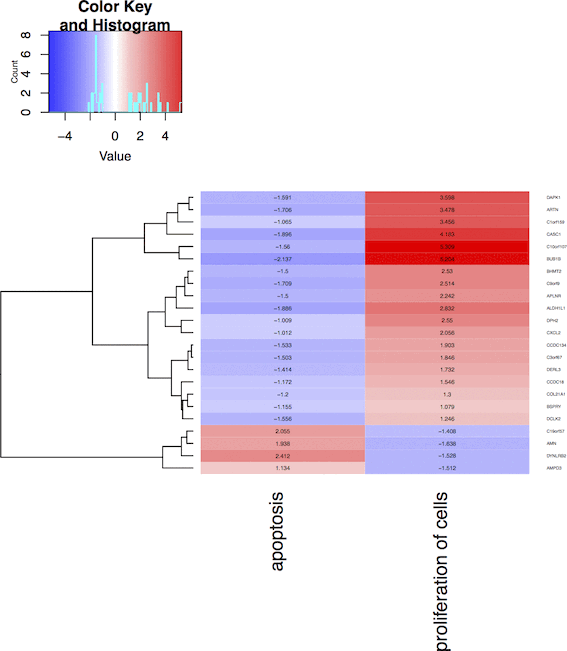

Supplement: S1 Data — (ZIP) [file pcbi.1012999.s005.zip › Moonlight2R-devel/vignettes/URAplot.gif]

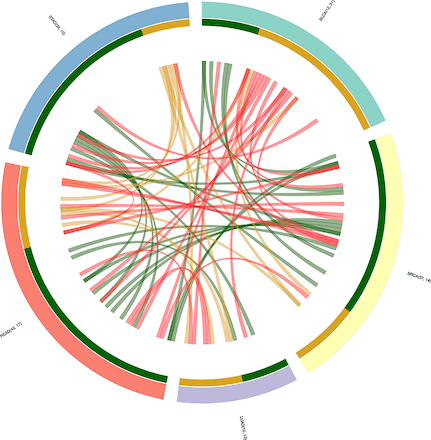

Supplement: S1 Data — (ZIP) [file pcbi.1012999.s005.zip › Moonlight2R-devel/vignettes/circos_ocg_tsg_ncancer5.gif]

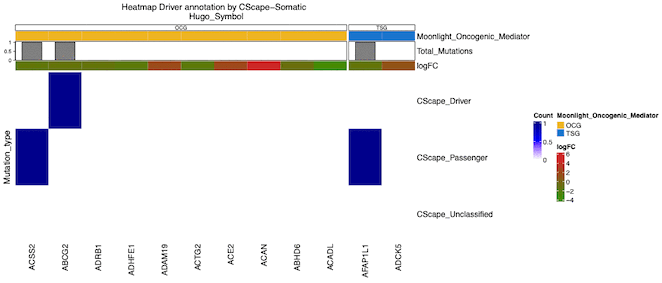

Supplement: S1 Data — (ZIP) [file pcbi.1012999.s005.zip › Moonlight2R-devel/vignettes/heatmap_complete.gif]

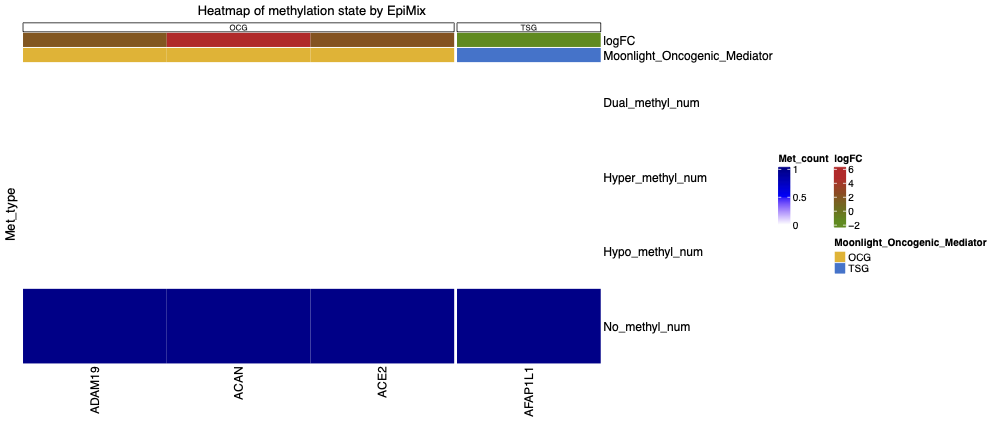

Supplement: S1 Data — (ZIP) [file pcbi.1012999.s005.zip › Moonlight2R-devel/vignettes/heatmap_genelist_met.png]

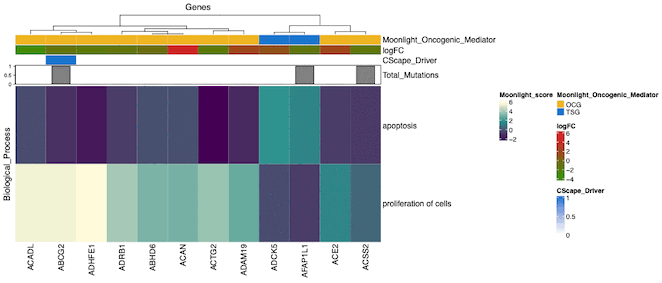

Supplement: S1 Data — (ZIP) [file pcbi.1012999.s005.zip › Moonlight2R-devel/vignettes/moonlight_heatmap.gif]

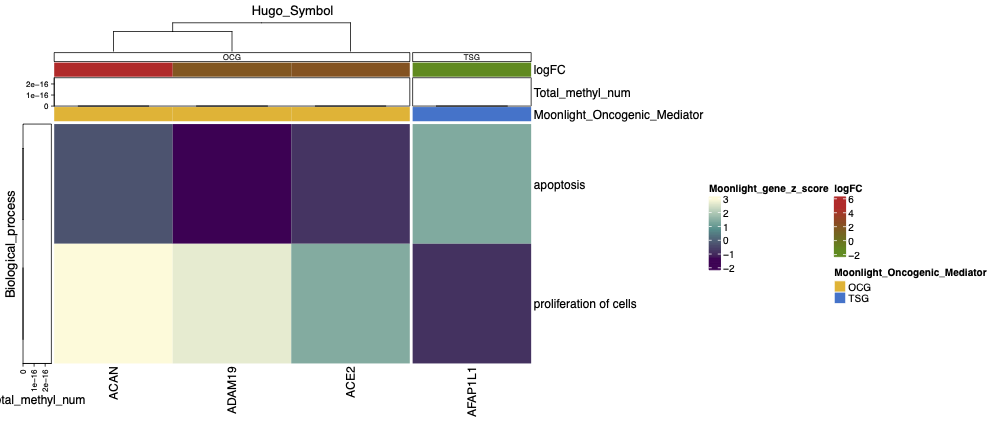

Supplement: S1 Data — (ZIP) [file pcbi.1012999.s005.zip › Moonlight2R-devel/vignettes/moonlight_heatmap_met.png]

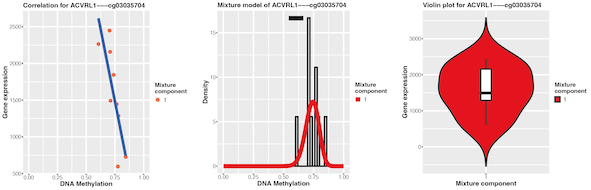

Supplement: S1 Data — (ZIP) [file pcbi.1012999.s005.zip › Moonlight2R-devel/vignettes/plotMetExp.png]
