# Supplementary material for: Revealing cancer driver genes through integrative transcriptomic and epigenomic analyses with Moonlight
Source: PLoS Comput Biol. 2025 Apr 21;21(4):e1012999. doi: 10.1371/journal.pcbi.1012999 (PMC12058160; doi:10.1371/journal.pcbi.1012999)
Supplement: Attachment — Submitted filename: Response_to_reviewers.pdf [file pcbi.1012999.s008.pdf]

We thank the reviewers for their insightful observations and questions. We have revised the manuscript and associated material according to their suggestions. Answers to the points they have raised can be found in the document below.

Reviewer 1

In this work, the authors presented an upgrade of their previously published software to predict driver genes for cancers. The main functionality incorporated in the upgrade is called Gene Methylation Analysis (GMA), which investigates abnormal DNA methylation patterns to predict driver genes. It was expressed clearly why such a functionality is in need.

However, the authors didn't put effort on describing the implementation of GMA. Without providing the detailed picture of GMA (e.g. the workflow/algorithm, and/or relevant mathematical expressions), the manuscript was not read very computational.

We thank the reviewer for their insights, we have added methodological details about Moonlight2R. EpiMix and GMA in the methods section, pages 5-9, trying to strike a balance between methodological accuracy and accessibility to a wider audience. More details on such changes are also available in our answers below.

A few addition comments:

1. *The authors applied GMA to three cancer (sub)types (basal-like breast cancer, lung adenocarcinoma, and thyroid carcinoma), and discovered 33, 190, and 263 epigenetically driven genes, respectively. the number of driver genes for basal-like breast cancer is of a much smaller magnitude compared to the other two types. Is it because a subtype of breast cancer was tested? If different breast cancer subtypes are tested, how do the driven genes overlap among subtypes?*

   We thank the reviewer for this insightful question. The much smaller number of predicted cancer drivers in basal-like breast cancer is indeed due to it being a subtype of breast cancer. We plan on investigating more in detail the differences and overlaps between breast cancer subtypes in a follow-up paper, which we are currently working on. We feel that investigating more cancer (sub)types is out of scope for this work, as this is a software paper showcasing the new functionality in Moonlight.

2. *Throughout the manuscript, sometimes the authors used term "functionalities", and sometimes "functionality". What are other functionalities added to Moonlight2 besides GMA?*

   We thank the reviewer for spotting this. We have now updated the text for consistency such that the term "functionality" is used which refers directly to the new GMA functionality. Accompanying the new GMA functionality, we have also created three functions for visualizing the GMA results (mentioned on page 10), which is why we sometimes used the

term “functionalities”; however, we believe that the term “functionality” is the best choice of wording.

3. *In the paragraph starting from Line 118, the authors brought an overview of the proposed frame, is built up on two layers. In what context is the term “layer” used? When I read “layer”, I thought it might be a neural network, and it’s not, so I got confused. I think the authors can phrase it more unambiguously and move the first cross-reference of Fig 1A up here.*

We thank the reviewer for bringing this to our attention. Indeed, the term “layer” does not refer to a neural network but rather to a conceptual framework consisting of two steps that need to be carried out. First the analysis on differentially expressed genes is performed by the primary step/layer, obtaining a list of putative driver genes, and then one or more secondary step/layer(s) need(s) to be used to provide a mechanistic explanation. We have now clarified the meaning of the term “layer” in the manuscript to avoid confusion for the readers. We have also moved the reference to Fig 1A up here. Please see the changes in the manuscript on page 5 lines 120-121.

4. *In Line 130, the authors said that adding functionalities to Moonlight2 was a challenge. However, the manuscript was not elucidated sufficiently why it is challenge. For example: in Line 229, the results from EpiMix and GMA were integrated? How is the integration preformed? Can this process be written algorithmally/mathematically? What were some difficulties during the integration?*
   4.1. *Please provide as many/detailed algorithms as possible so that manuscript would fit Computational Biology.*

We appreciate the reviewer’s feedback. Our intention with the phrasing: “In this contribution, we tackled the challenge of adding functionalities to Moonlight2 to cover mechanistic indicators related to methylation changes” was not to highlight the difficulty of adding functionalities to Moonlight2 but instead to describe the process of expanding Moonlight2 to include another mechanistic indicator related to methylation changes. To avoid misunderstandings, we have now revised this sentence for clarity: “In this contribution, we added another secondary layer to Moonlight2 to cover mechanistic indicators related to methylation changes”. The updated sentence is included on page 7 lines 149-150.
We would like to clarify that the results from EpiMix and GMA are not integrated, indeed results from EpiMix are used as a source of information and are fully contained in the GMA secondary layer. Instead, the results from EpiMix and PRA (Moonlight’s primary layer) are integrated, using GMA. We have now added a more detailed explanation of how this integration is performed, which also includes a new table (Table 1) that shows the integration process. These new changes are included on page 8-9 lines 188-202.

5. *How were the cancer types chosen in this work?*

We selected the cancer types because we also analyzed these cancer types in our previous paper where we introduced the DMA (mutational) functionality to Moonlight. Continuing with the

same cancer types allowed us to compare the results and build upon our previous findings. For clarification, we have now added a sentence in the manuscript with this reasoning. This has been added to the manuscript on page 11 lines 253-255.

6. *In Fig 4B-4D, although the p values are statistically meaningful, the absolute difference between high and low expression patient groups are close. In particular, in Fig 4D it seems that high-expression group can live longer (though with a slightly smaller probability) than low-expression group. Given Fig 4B-4D only I don't think the findings in Fig 4A are convincing.*

We thank the reviewer for the insightful comment. We agree with the reviewer that the absolute difference between high and low expression patient groups are close in Fig 4B-4D. While the Cox regression analyses did show significant effects on survival of 22 oncogenes, the magnitude of survival differences from Kaplan-Meier analyses are small which can limit the clinical potential. We have now discussed these implications in the manuscript. These changes can be found on page 18 lines 438-445.

7. *In Fig 2E, could you change a way of visualization? Make the metrics x-axis and the functionalities the hue category. It's very hard to tell from the current plot which functionality outperforms under a certain metric.*

We thank the reviewer for the suggestion. We have now changed Fig 2E to reflect metrics on the x-axis (precision, sensitivity) and functionalities with the hue category (GMA, Moonlight primary layer). This highlights which functionality outperforms the other for a specific metric.

8. *In Fig 4A, no genes were from breast cancer. Why not?*

This is because none of the breast cancer driver genes were found to be prognostic from our survival analysis. Fig 4A only includes driver genes which we found to be prognostic. To make this more explicit, we have now added a sentence in the paragraph describing Fig 4A that no driver genes in breast cancer were found to be prognostic. This has been added on page 17 lines 453-455 in the manuscript.

Reviewer #2

This manuscript presents an extension of the publication of moonlightR (Nourbaksh et al. 2023, 10.1093/bib/bbad274) where the utility of driver mutation analysis incorporating Cscape-somcatic was first introduced. In brief, moonlight has two layers. In the first layer, differential expression analysis yields candidate genes which are scored through several means and then partially labeled as oncogenic mediators. In a second layer, DMA is then applied, which essentially means that we look if there is a known driver mutation in the gene as I understood it. In this work, an alternative secondary layer is added which leverages the Bioconductor package EpiMix to identify DNA methylation changes that are linked to expression changes. This new functionality is termed gene methylation

analysis (GMA) and results in a list of CpGs associated with expression changes and methylation state (hyp- or hypermethalyted).

The manuscript is mostly well written but focuses mainly on technical details and to a lesser extent on methodological details needed to understand the method fully. Source code for the application case as well as the package itself is freely available on github. I feel that the added value of integrating methylation information in the package through EpiMix is only an incremental addition compared to the previous version of moonlight described in last year's paper. I have the following specific comments:

# Major

- The principles of DMA and PRA are insufficiently described in this manuscript and can only be understood if the reader is familiar with the previous manuscripts. Please offer a more accessible introduction to moonlight's concept for readers hearing about moonlight for the first time.

We thank the reviewer for this suggestion of how to make the manuscript more readable and understandable. We have now added a more detailed and thorough description of the principles behind Moonlight in the beginning of the manuscript. These changes are added on pages 5-7 lines 121-158.

- The manuscript is vague on how the results of DMA and GMA are integrated. More information is found in S1 Text, which definitely needs to be integrated into the main manuscript to make this approach understandable . The description of the integration could nevertheless be a bit clearer. For example, what happens with the results that are not in agreement between DMA and GMA? Surely one of these layers is sufficient to determine a potential oncogene or tumor suppressor. Methylation and mutation information do not have to be always in agreement, there are ample examples where this is not the case.

We thank the reviewer for the comment. We would like to first clarify that the results of DMA and GMA are not explicitly integrated in our pipeline. It is the results from EpiMix and PRA (PRA is from Moonlight's primary layer) that are integrated which constitutes the principle of the GMA functionality presented in the manuscript. DMA is another functionality in Moonlight that we have previously published and have referenced in this current manuscript. The principle behind GMA is to integrate gene expression changes with DNA methylation alterations (hypo- or hypermethylation status), whereas the principle behind DMA is to integrate gene expression changes with driver mutations. The results of DMA and GMA are not integrated because, as also pointed out by the reviewer, methylation and mutation information are not always in agreement and can be seen as complementary. DMA and GMA are meant as two separate secondary layers in Moonlight that are not dependent on each other such that it is up to the user to apply either DMA or GMA (or both) depending on what data the user has available and their research question at hand. Thus, DMA and GMA are meant to complement each other as we have also showcased in the case study where we are comparing the results between DMA and GMA. On page 7, we had stated that the secondary layers can be applied together or separately depending on the available data, however, we have now refined this sentence to make it clearer. These changes have been added on page 7 lines 153-158.
Moreover, we have now integrated the text from “S1 Text” into the main text to make the integration between EpiMix and PRA more explicit. These changes have been added on pages 8-9 lines 188-202.

- EpiMix is not a published or evaluated approach and insufficient details are provided here to understand how EpiMix is used. Also, the working principles behind EpiMix should be explained in more detail as this is the novel feature added in this manuscript. For example, are only promoter regions considered here? How are CpGs generally linked to genes?

EpiMix is indeed a published method which we have referenced in the manuscript, please see e.g. line 165 in the manuscript and reference 21, “Zheng Y, Jun J, Brennan K, Gevaert O. EpiMix is an integrative tool for epigenomic subtyping using DNA methylation. Cell Reports Methods. 2023;3: 1–23. doi:10.1016/j.crmeth.2023.100515”. EpiMix has been developed by co-authors of the manuscript (Yuanning Zheng and Olivier Gevaert) and builds upon a previously published method called MethylMix (10.1093/bioinformatics/btv020, 10.1093/bioinformatics/bty156). We would like to refer the reader to the EpiMix paper for a detailed description to how EpiMix works, however, we have now added a brief introduction to EpiMix in the manuscript for clarity. Please see the changes on pages 7-8 lines 169-175.

- In the use case, the authors highlight that the identified driver genes are potential therapeutic drug targets but as far as I see this analysis is not a part of moonlight's functionality and thus somewhat unrelated. The authors may think about integrating such functionality.

The analysis exploring the therapeutic potential of the identified driver genes is to demonstrate a potential post hoc application of the driver genes predicted by Moonlight. We believe such an analysis falls outside the scope of Moonlight's core functionality, which is specifically designed to predict driver genes. Instead, by demonstrating potential post hoc applications such as survival analysis, therapeutic potential etc, we encourage users to use Moonlight's predictions in their own downstream analyses tailored to their research interests. We have also shared all the code used in our case study on GitHub which users can reuse to perform similar analyses.

- The application case should highlight more clearly if there was any added value of considering methylation data. Beyond a tradeoff of specificity / sensitivity w.r.t. COSMIC genes, how are the new results more relevant to the users. What new hypotheses could be generated here? The authors focus more on commonalities than on differences and unique insights which would be also relevant.

We thank the reviewer for the suggestion. We have now added a paragraph in the manuscript discussing how the new results are relevant and what new hypotheses can be generated moving forward. We have added these new changes on pages 23-24 lines 575-584.

# Minor

- Supplemental material does not give information about package or software versions used for the analysis.

We have included information about package and software versions used for the analysis in a renv.lock file in the GitHub repository which includes our case studies (https://github.com/ELELAB/Moonlight2_GMA_case_studies/blob/main/renv.lock), as we believe GitHub is the most appropriate platform to disseminate such information. Renv is a package designed to create, handle and share reproducible computational environments, and a renv.lock file carries the information necessary to rebuild such an environment, explicitly including all used packages and their versions. In the GitHub repository we also include detailed instructions on how to reproduce the computing environment as well as our calculations.

- I think the comparison of DMA and GMA results could be supported by Venn diagrams.

We have included Venn diagrams that visualize the comparison of the DMA and GMA results in Figure 6.

Reviewer #3

This manuscript proposed a tool called Moonlight2, which introduced a layer of Gene Methylation Analysis (GMA) to predict the cancer driver genes such as oncogenes and tumor suppressor genes. Moonlights integrates both DNA methylation data and transcriptomic data. This research further applied the GMA on three cancer types, including basal-like breast cancer, lung adenocarcinoma, and thyroid carcinoma. It showed that these epigenetically driven genes have the potential to forecast the prognosis of cancer patients as well as predict drug targets.

Overall this work seems to be innovative by including additional methylation data, which is important but less explored. The methods are clearly described, including the integration of EpiMix and CpG-gene pairs in methylation status. The figures are presented clearly and precisely. The analysis of genes such as PDGFRB and MET seems to be compelling to be considered as potential drug targets. Finally, the code and data are open sourced and publicly available.

The manuscripts may benefit by addressing the following concerns:

- The manuscript does not have a "discussion" section, which may mention the limitations of the research, such as the lack of experimental validation of key driver genes.

We agree with the reviewer and had initially considered adding a discussion section, however, unfortunately, such section is not part of the Software paper format according to the PLoS Computational Biology, as per their guidelines (https://journals.plos.org/ploscompbiol/s/submission-guidelines#loc-software-submissions). However, we have now added a sentence in the "future directions" section that mentions that experimental validation of the driver genes will be needed in the future. This has been added on page 24 line 613.

- Relevant to the point above, it will be helpful to further discuss the difference (specifically pros/cons) of using methylation-driven predictions of driver genes, in comparison to mutation-driven predictions of driver genes.

We thank the reviewer for the great suggestion. We have now added a paragraph in the manuscript that discusses the difference (pros/cons) of using methylation- vs mutation-driven predictions of driver genes. These changes have been added on page 23 lines 566-574.

- This research mainly focused on three cancer subtypes. Expanding the study to include a pan-cancer analysis would strengthen a broader applicability of the tool.

We appreciate the reviewer's suggestion to include a pan-cancer analysis as this indeed would showcase a broader applicability of the tool. However, we believe that this is beyond the scope of the current manuscript which is a software submission focusing on presenting a new method within Moonlight. Our goal is thus to introduce the tool and demonstrate its functionality through case studies. We believe that a comprehensive pan-cancer analysis would be better suited in a follow-up study that explores specific biological questions. Before engaging in such a study, it was a prerequisite for us to have the new method presented in the current manuscript.
