# Supplementary material for: Revealing cancer driver genes through integrative transcriptomic and epigenomic analyses with Moonlight
Source: PLoS Comput Biol. 2025 Apr 21;21(4):e1012999. doi: 10.1371/journal.pcbi.1012999 (PMC12058160; doi:10.1371/journal.pcbi.1012999)
Supplement: Attachment — Submitted filename: PCOMPBIOL-D-24-01980 comments.docx [file pcbi.1012999.s006.docx]

In this work, the authors presented an upgrade of their previously published software to predict driver genes for cancers. The main functionality incorporated in the upgrade is called Gene Methylation Analysis (GMA), which investigates abnormal DNA methylation patterns to predict driver genes. It was expressed clearly why such a functionality is in need. However, the authors didn’t put effort on describing the implementation of GMA. Without providing the detailed picture of GMA (e.g. the workflow/algorithm, and/or relevant mathematical expressions), the manuscript was not read very computational.

A few addition comments:

1. The authors applied GMA to three cancer (sub)types (basal-like breast cancer, lung adenocarcinoma, and thyroid carcinoma), and discovered 33, 190, and 263 epigenetically driven genes, respectively. the number of driver genes for basal-like breast cancer is of a much smaller magnitude compared to the other two types. Is it because a subtype of breast cancer was tested? If different breast cancer subtypes are tested, how do the driven genes overlap among subtypes?
2. Throughout the manuscript, sometimes the authors used term “functionalities”, and sometimes “functionality”. What are other functionalities added to Moonlight2 besides GMA?
3. In the paragraph starting from Line 118, the authors brought an overview of the proposed frame, is built up on two layers. In what context is the term “layer” used? When I read “layer”, I thought it might be a neural network, and it’s not, so I got confused. I think the authors can phrase it more unambiguously and move the first cross-reference of Fig 1A up here.
4. In Line 130, the authors said that adding functionalities to Moonlight2 was a challenge. However, the manuscript was not elucidated sufficiently why it is challenge. For example: in Line 229, the results from EpiMix and GMA were integrated? How is the integration preformed? Can this process be written algorithmally/mathematically? What were some difficulties during the integration?
   4.1. Please provide as many/detailed algorithms as possible so that manuscript would fit Computational Biology.
5. How were the cancer types chosen in this work?
6. In Fig 4B-4D, although the p values are statistically meaningful, the absolute difference between high and low expression patient groups are close. In particular, in Fig 4D it seems that high-expression group can live longer (though with a slightly smaller probability) than low-expression group. Given Fig 4B-4D only I don’t think the findings in Fig 4A are convincing.
7. In Fig 2E, could you change a way of visualization? Make the metrics x-axis and the functionalities the hue category. It’s very hard to tell from the current plot which functionality outperforms under a certain metric.
8. In Fig 4A, no genes were from breast cancer. Why not?
